# The influence of sex in diagnostic modelling of knee osteoarthritis

**DOI:** 10.1371/journal.pone.0325681

**Published:** 2025-07-03

**Authors:** Philippa Grace McCabe, Paulo Lisboa, Bill Baltzopoulos, Ian Jarman, Kellyann Stamp, Ivan Olier

**Affiliations:** 1 School of Pharmacy and Biomolecular Sciences, Liverpool John Moores University, Liverpool, England, United Kingdom; 2 Data Science Research Centre, Liverpool John Moores University, Liverpool, England, United Kingdom; 3 School of Computer Science and Mathematics, Liverpool John Moores University, Liverpool, England, United Kingdom; 4 Research Institute for Sport and Exercise Sciences, Liverpool John Moores University, Liverpool, England, United Kingdom; 5 Liverpool Centre for Cardiovascular Science, University of Liverpool, Liverpool John Moores University and Liverpool Heart and Chest Hospital, Liverpool, England, United Kingdom; University College London, UNITED KINGDOM OF GREAT BRITAIN AND NORTHERN IRELAND

## Abstract

**Objective:**

To compare diagnostic models for radiological KOA at KL2 + using sex-specific variables against a generic model with sex as an input. Data from the Osteoarthritis Initiative (OAI) was used for model development and optimisation.

**Materials and methods:**

Current models for diagnosis of knee osteoarthritis (KOA) at first presentation comprise subjects in the OAI dataset with and without KOA. We select subsets of the OAI data set for which additional sex-specific variables are available, resulting in male and female cohorts of size n = 1250 and n = 1442, respectively.

**Results:**

The classification performance of the previous diagnostic model on the test data has an area under the curve (AUC) of (95% CI 0.721–0.774) when only variables common to both sexes were entered for model selection and sex was a separate input. When tested separately on the male only and female cohort the test performance of the generic model gives baseline AUCs of (95% CI 0.689-0.770) and (95% CI 0.728-0.799) respectively. The sex-specific models for males and females yield AUCs of (95% CI 0.684-0.765) and (95% CI 0.731-0.803) respectively,

**Discussion:**

Fitting sex-specific models allows additional variables to be entered in the pool for model selection compared with a generic model with sex as a covariate. The focus of this study is whether the specificity of the additional data enhances their predictive power of logistic regression modelling for the diagnosis of incident radiological KOA in the OAI dataset, at first presentation. The performance of the generic and sex-specific models is comparable, since the confidence intervals for all of the models overlap. Nevertheless, some relevant variables after feature selection v are sex-specific, indicating that incidence of KOA at baseline presentation is associated with sex-specific attributes.

**Conclusion:**

This specialisation of the sex-specific models indicates potential differences in the aetiology leading to disease onset and may provide greater utility to both clinicians and subjects. For instance, the risk factors identified by the specialised models provide quantitative indicators that useful for early identification of females at higher risk of KOA, prompting them to take proactive measures to improve joint health at an earlier stage in life.

## Background and significance

Women, constituting approximately half of the global population, often face neglect in various fields, including medicine, where their specific needs are disregarded, reflecting historical biases traced back to ancient times [1]. Despite advocacy for the inclusion of females in clinical trials and sex-based analysis, such efforts remain inconsistent [2–6]. The Women’s Health Equity Act of 1993 marked a pivotal shift by granting women access to medical studies previously unavailable to them, shedding light on conditions predominantly affecting female health [7,8]. Discrepancies in pain treatment between sexes persist, with women frequently facing dismissal or misdiagnosis due to varied disease presentations and psychogenic symptoms [9–13].

Sex disparities extend beyond clinical care, impacting disease manifestation, immune responses, and medication effectiveness [14–23]. From differing heart mechanics influencing heart attack presentations to heightened autoimmune responses in females, biological variations underscore the necessity for tailored medical approaches [16–21]. Despite these findings, sex-neutral dosing practices persist, perpetuating risks of adverse reactions and overdosing, particularly among women [4,22]. Beyond medicine, sex biases permeate various domains, including the design of personal protective equipment and workplace environments, reflecting systemic challenges stemming from skewed data representation and model training [23]. Examples of this type of sex bias include how PPE is made, by treating the female outline as a small male and failing to consider physiological and anatomical differences, and the average office temperature, with females ‘suffering’ in both of these scenarios. In many cases, the models that are used in medical settings have been trained in unevenly represented populations, with the demographics of the data leaning toward male dominance.

It is known that the prevalence, incidence and severity of osteoarthritis (OA) differ between males and females [23], with females more likely to suffer than men in each of these aspects [24]. females aged 50–60 may be 3.5 times more likely to develop OA of any type than males in the same group [25]. Moreover, females are 40% more likely to develop knee osteoarthritis (KOA) than men [26]and are also more likely to experience more severe symptomatic KOA [24,27]. Typically, when females present to a clinician with OA they are in the advanced stages of disease and are suffering from more debilitating pain [28]. It has been noted for many years that there are significantly higher number of females with symptomatic disease than their male counterparts [29]. However, this knowledge has not triggered a study to investigate potential biomarkers that could explain the difference in presentation and development of the disease [28,30].

Although the exact reasons for these differences are not yet fully understood, some speculation exists regarding the underlying causes [31]. Hanna et al. [32] suggests that hormonal differences, in addition to anatomical factors between males and females, may play a significant role in the manifestation of the disease. Variations in bone density and size, likely influenced by hormonal factors, could contribute to the distinct development patterns of KOA in males and females. Research indicates that at baseline, females are more likely to have a greater number of cartilage defects and experience accelerated cartilage loss compared to males. This hormonal influence may result in a higher risk of cartilage defects in females, a known risk factor and indicator of KOA [33].

Kinematic differences between males and females may also contribute to the development of KOA in females [28,34–38]. Female athletes place additional stress on their joints, which can increase their risk of developing KOA [28]. Furthermore, hormonal differences between males and females—such as fluctuating levels of oestrogen and progesterone—can affect ligament laxity and joint stability, making females more prone to joint injuries, including anterior cruciate ligament (ACL) tears. Female knees also have musculoskeletal differences that influence joint loading and wear [39,40].

In addition to this, the way that injuries are managed and followed up can also contribute to the development of KOA [41]. While damage to the anterior cruciate ligament (ACL) can lead to osteoarthritis (OA) in both males and females, females are more likely to experience ACL injuries due to both hormonal and biomechanical factors [28,42,43]. Oestrogen, for example, is believed to affect collagen synthesis and joint elasticity, increasing the susceptibility of ligaments like the ACL to damage. A study by Chu et al. suggests that early intervention after ACL injuries may have the potential to reduce the development of arthritis in both sexes, particularly in females who are at greater risk [41].

The pathogenesis of KOA involves the progressive degeneration of articular cartilage, leading to joint space narrowing, subchondral bone remodelling, and inflammation. Contributing factors such as obesity and previous joint injury exacerbate mechanical stress on the knee [44], accelerating cartilage breakdown and increasing the risk of disease progression [45]. Another feature that has a significant role in the pathogenesis of KOA is the infrapatellar fat pad (IFP). IFP is influenced by sex and higher levels suggest an increased risk for developing KOA. It has been noted that there is an increase of IFP volume in healthy males compared to healthy females, suggesting an increased risk of KOA for males [46,47]. However, even when considering this, after factoring in for hormonal changes, females are at a higher risk of KOA.

Among the other factors that contribute to the onset of KOA, hormone levels have a role in disease development. A females susceptibility to OA may be related, in part, to hormone levels [48]. After the menopause, when oestrogen drops, females are at an increased risk of developing OA as cartilage and joint health is impacted. This has been linked to a drop in oestrogen, as there are oestrogen receptors present in cartilage, where decreased oestrogen levels may contribute to an increase in cartilage breakdown as oestrogen receptors in joint tissues support cartilage health [49].

As the focus of this paper is to develop models that are sex specific, variables that account for hormonal changes a person may experience are included in the analysis. The sex specific variables in this work include male specifics -- accounting for the use of testosterone or having a diagnosis of prostate cancer. Female specific variables including whether the subject has had a hysterectomy, had ovaries removed, been pregnant and/or if they have had hormonal treatment for menopause or bone density issues. The rationale for this is that in both cases, hormone levels will have been varied for a period of time, potentially leading to altered risk of developing KOA.

The work in this paper builds on the existing models that have been successfully validated by the Multicenter Osteoarthritis Study (MOST) data, described McCabe et al. [50]. By considering the model without sex as a variable we can establish whether a global model, with and without sex, is suitable for the whole population, or if there is a benefit for considering male and female subjects separately, with sex specific variables available for inclusion into the model.

The work in this paper looks to follow on from previous analysis into the features that indicate KOA in both males and females [50], and using that model as a benchmark, aims to further investigate the role of sex specific features to assess the impact on performance for a diagnostic model in each cohort.

### Aim of this work

• To determine whether the introduction of variables specific to each sex alters the performance of the diagnostic model for both the male and female cohorts.

## Materials and methods

### Study design

Although previous work has already made considerations towards adjusting for the effect of sex [46], this work does not consider the contribution of sex-specific covariates, as detailed in Table 1. When looking at how best to implement the models to consider sex, two approaches became apparent. The first approach mirrors that used which was used in the paper by McCabe et al. [50]. This is to take the original pool of variables, which are common to subjects of both sexes, and build a separate model for each sex. The model will then use the respective data test sets for the total and individual male and female cohorts, to assess performance.

**Table 1:** A table describing the sex specific variables included in the models, the data label, and the definition of each variable. Each variable mentioned in the table has a binary outcome, either yes or no.

*Sex*	Variable Name in Dataset	Definition
Male	P02CNC13	Has the subject got or previously had prostate cancer?
	V00TEST	Has the subject used male hormones in the previous 6 months (prior to initial visit)?
Female	P02HYS	Has the subject had a hysterectomy?
	P01OVREM	Has the subject ever had an ovary removed?
	P01PREGEV	Has the subject ever been pregnant?
	V00ESTR	Has the subject ever used a combination of oestrogen and testosterone as either treatment for menopause or to increase bone density?

The second approach starts the same as the first, by splitting the data pool by sex. The second approach also splits the data pool by sex, but additionally adds the sex specific variables to the respective set. On each of the expanded variable sets, follow a feature selection protocol to determine the most influential variables. For each sex then build a model using the selected features. Assess the individual model performances and compare these to the original model. This will also highlight if the inclusion of sex specific variables is beneficial when considering males and females separately for predicting the presence of KOA.

### Data – Osteoarthritis Initiative (OAI)

For the modelling and validation in this paper, we have used data from the Osteoarthritis Initiative (OAI). The OAI knee health study is a multi-centre longitudinal study that aimed to understand the development and progression of KOA.

The OAI was conducted over a 10-year period in America starting in 2004, initially made up of 4796 subjects, aged 45–79 who were recruited based on their likelihood to develop KOA [51]. In the OAI study, clinical examinations, questionnaires and telephone interviews were conducted at varying intervals and the results recorded. In the analysis described in this paper, the data that is used is collected from questionnaires that the subject completed, and the Kellgren-Lawrence (KL) score, used as the indicator for presence of KOA. This information is collected from the clinician analysing the x-rays. The KL score is a widely used system to classify the severity of OA based on x-rays after a clinician has evaluated them for the presence of features indicating disease. For the variables used in the models in this analysis only data recorded at the initial visit was required as inputs into the model and the diagnostic KL grade as the outcome variable. In this analysis, a KL score of grade 2 or greater is classified as presence of radiographic KOA, and a grade of 0 or 1 indicates no disease. Researchers and clinicians agreeing that KL2 + constitutes the presence of radiographic KOA [29].

The raw data tables used in these analyses from the OAI are named AllClinical00, which combines data about subject characteristics, risk factors and medical history, and kxr_sq_bu00, is the data table that which contains information relating to the clinically assessed x-rays at baseline with the relevant KL grades. The main variables are taken and adapted from AllClinical00, whilst the outcome for the diagnostic model is from kxr_sq_bu00. The data is joined using the subject ID to merge the relevant rows to contain the data needed for the diagnostic model.

### Sex specific factors

In both the diagnostic and prognostic models proposed by McCabe et al. [50], the variable Sex was found to be statistically significant. In the prognostic model, the majority of the high-risk cohort was female.

Several sex specific variables were identified and extracted from the OAI data. The original cohort was then split by sex and the additional variables added to each cohort respectively.

The female specific variables include a history of; hysterectomy, oophorectomy, pregnancy or hormonal treatment for menopause or bone density issues. The male variables consider the use of testosterone in the six months prior to data collection, and history of prostate cancer. The variables added to the models for male and female are described in Table 1.

The OAI data contained other variables that could be of interest when considering their impact in relation to sex and the onset of KOA, however, these variables were seen as either not relevant in a sex-separated study (i.e., affect both sexes, such as breast cancer) or did not provide sufficient information to the model (such as grouped cancer diagnoses). Data relating to the diagnosis of cervical cancer has also been removed from the dataset – cervical cancer can only affect people with a cervix and therefore, if included, would be considered in the female modelling only. However, in the OAI data for the original cohort, all subjects have ‘NA’ values, rendering this variable unusable.

The final variable that could be of interest in the sex specific analysis is how many ovaries have been removed. For this reason, the binary version of this variable ‘Have you had ovaries removed?’ was used instead of ‘How many ovaries have you had removed?’. The latter option could provide an initial step for future investigation into what, if any, difference to disease presence occurs as a result of removing one, both or no ovaries. A subsequent step could be to explore the impact of the type of hysterectomy a person has in relation to KOA, as there are three types, with only one resulting in the removal of the ovaries.

**Fig 1:**
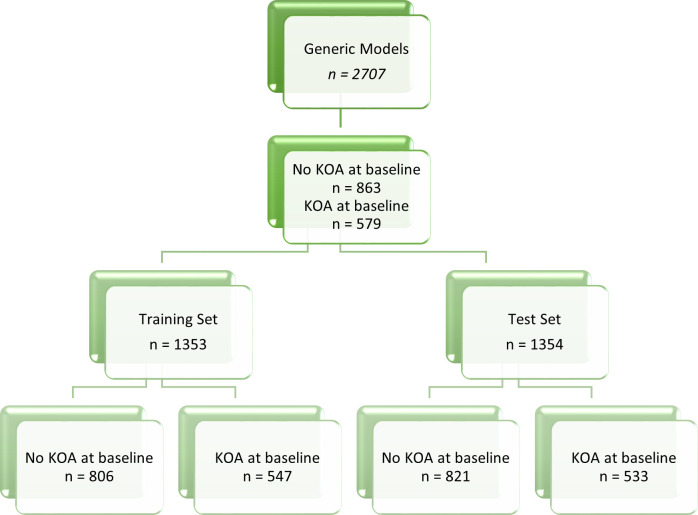
Data breakdown of whole data cohort detailing prevalence of KOA and the training and test set sizes. This flow diagram shows the data breakdown for the generic data model.

**Fig 2:**
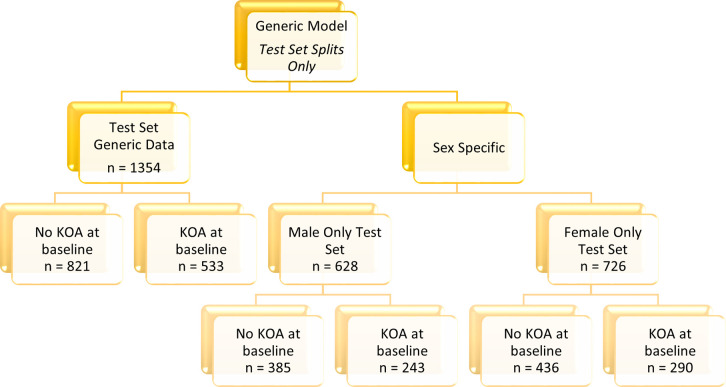
Data breakdown of test data cohort detailing prevalence of KOA and test set sizes. This diagram shows the test set breakdown for the generic test data and the female and male only cohorts tested on the generic model.

**Fig 3:**
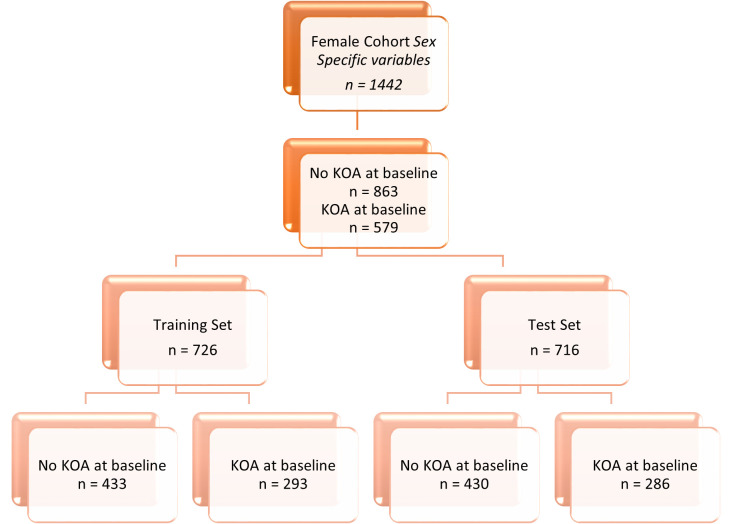
Data breakdown of the female data cohort detailing prevalence of KOA and the training and test set sizes. This diagram shows the female cohort for the sex specific variable approach.

**Fig 4:**
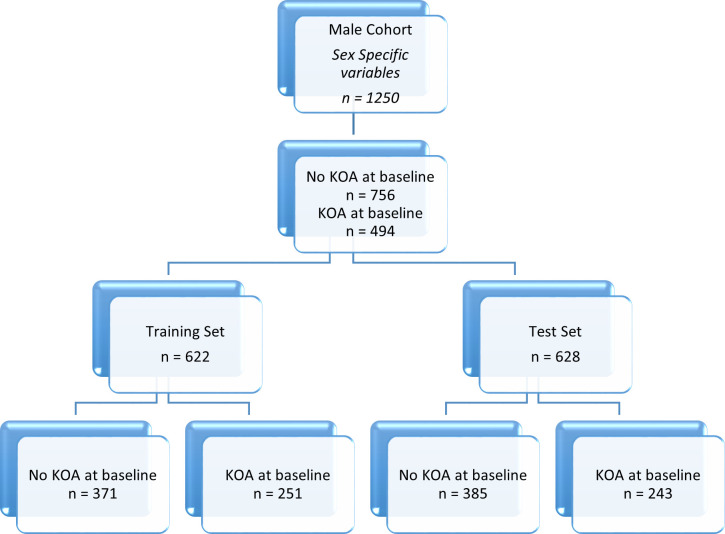
Data breakdown of the male data cohort detailing prevalence of KOA and the training and test set sizes. This diagram shows the male cohort for the sex specific variable approach.

The data in this paper is specifically grouped by sex for the analysis, resulting in two cohorts: male and female. In each of these cohorts there are training and test splits to allow the model performance to be assessed out of sample. The male cohort consists of 1250 subjects, with the prevalence of disease, determined as having KL2+ after clinical review of radiographs, at 40% of the participants. The female cohort varies by study approach, due to some subject having missing values for the variables of interest. The original variable approach gives a female cohort size of 1457, and the inclusion of sex specific variables reduces the cohort to 1442 subjects. The way in which the data is split for modelling and testing is detailed in Figs 1–4.

### Data pre-processing

The data processing in this work follows the same format as when designing the diagnostic and prognostic models [46]. The OAI dataset contained a sample of 4796 participants, after removing individuals without KL scores a sample of 4507 subjects remained. Following a complete case analysis resulted in a cohort of 2707 subject.

This analysis uses a comprehensive dataset that integrates clinical factors, demographic information, self-reported symptoms, and physical activity data. The clinical and demographic variables include age, gender, and body mass index (BMI), as well as details about family history and previous knee injuries. Self-reported symptom data were collected through questionnaires administered during the initial visit, capturing how patients experience and are affected by their symptoms. Similarly, information on physical activity was gathered from self-report questionnaires detailing exercise frequency and its impact on the individuals.

The cohort considered in this analysis was only subjects without any missing values for the selected variable set, as a complete case analysis [52]. The original OAI full data cohort had a sample size of 4796. After removing those who have no KL grade, a sample of 4507 subjects remained. Finally, removing those subjects who have missing values in any portion of the variable sets used in the analysis leaves a usable cohort of 2707 subjects in the complete cohort.

## Implementation

### Experimental set-up

In this study, Akaike Information Criterion (AIC) was utilized to optimize logistic regression on the test data. Within the context of logistic regression, AIC balances the fit and complexity of the model in the process of model development. Stepwise feature selection using AIC was used to include only variables that are beneficial to the model, helping to limit the number of features in the model [53]. The entire process, including data pre-processing, analysis, and development of the application, was conducted in the R programming language [54]. The logistic regression model utilized the built-in functions for analysis available in base R.

In the logistic regression model a threshold for classification is determined, for values that fall below are given one class, with values above given the other. The threshold is typically determined using the prevalence of the feature in the population. As the prevalence in the dataset for individuals who suffer from KOA is between 45 and 60%, the classification threshold in these models are set to 0.5. A prediction less than or equal to 0.5 will render no KOA, with a probability greater than 0.5 will result in a decision of KOA.

### Measure of performance

The receiver operating characteristic (ROC) curve is a visual way to show how well a model can correctly classify two outcomes, such as whether a person has a disease or not, as the threshold for making decisions is changed. The area under the curve (AUC) gives a number that tells how accurate the model is; a value of 0.5 indicates that the model is performing no better than random chance, while a number higher than 0.5 means the model is more efficient [55]. To calculate the AUC and its confidence levels, the pROC package is used [56], applying a method that breaks the curve into sections and repeats the calculation many times to get an estimate [57]. The pROC package is an R package used to display and analyse ROC Curves [56].

In binary classification tests, sensitivity, specificity, and positive predictive value (PPV) are all statistical measures of performance. Sensitivity shows how well the model identifies true positive cases, while specificity determines the proportion of actual negatives that are correctly identified. PPV is a measure of the number of correctly classified subjects out of the entire group of disease class predictions, showing how accurate a positive result is. These measures are computed in this study using the caret package [58].

Whilst other performance metrics, such as F1 score, accuracy and negative predictive value are useful, for research with medical implications, sensitivity, specificity, and PPV are preferred as they provide clearer, more actionable information about how well a diagnostic model performs in real-world scenarios [59,60].

### Diagnostic model

The model used in the diagnostic analysis was logistic regression. The logistic regression approach was chosen after considering alternative analysis methods [61]. The logistic model aims to use several subject-related features to determine the likelihood of KOA and further investigation of symptoms. The number of features used in the model is dependent on the modelling approach employed. The outcome of interest is the presence of radiographic KOA, specifically KL grade 2 or higher. For male-specific models, original and sex-specific variables were used to train and test 622 and 628 subjects respectively, using OAI data. Female-specific models utilized original variables to train and test 731 and 726 subjects respectively, and a combination of original and sex-specific variables to train and test 726 and 716 subjects, respectively.

The models for both female and male cohorts were built using stepwise feature selection using AIC to determine which combination of variables to include. Following the use of the AIC measure the female model uses the variables age, BMI, baseline symptoms, knee swelling, limiting knee stiffness, history of pregnancy and history of hysterectomy. Two of the four female-specific variables were chosen by feature selection to be included in the model. Although neither of these were calculated to be significant in the model, they still contributed to the prediction. Similarly, following the same process, the variables in the male-specific model were age, BMI, baseline symptoms, knee swelling, limiting knee stiffness, difficulty getting upstairs and history of prostate cancer. One of the ‘male’ variables was chosen by feature selection and therefore included in the model for men.

## Results

The data in Table 2 show a summary of the models used, and their performance values, to assess the most beneficial way to incorporate sex into diagnostic modelling. Row 1 shows the results for the generic model, which serve to benchmark the model with sex as in previous work [50]. When testing the generic model on the male and female cohorts individually, there is a bigger difference in AUC performance compared with the generic model for females than that for males. Moreover, a McNemar test comparing the predictions for each sex specifically shows clear statistical significance for females (p = 0.005). This confirms that the female sex specific model performs better in the cohort after including additional sex specific variables.

**Table 2:** A table of performance metrics for the different variable sets used for the analysis, giving the area under the curve (AUROC), sensitivity, specificity, and positive predictive value (PPV).

		AUROC (CI)	Sensitivity	Specificity	PPV
1	Generic Model	0.748 (0.721–0.775)	0.520	0.848	0.773
2	Generic Model predicted for Female only	0.764 (0.728–0.799)	0.514	0.874	0.819
3	Generic Model predicted for Male only	0.729 (0.689–0.770)	0.527	0.821	0.746
4	Female Sex Specific Model	0.767 (0.731–0.803)	0.490	0.898	0.827
5	Male Sex Specific Model	0.725 (0.684–0.765)	0.482	0.821	0.729

All models consistently produce high specificity values, indicating the models suitability for identifying cases where KOA is not present. In each case where there are male and female specific models, the female model outperforms that of the male. This is a clear indication that there is a need for sex-disaggregated modelling within medical applications.

Looking at the results in Table 2 it is clear that there is a small amount of variation in model performance. Consistently, the best performing models are those trained and tested on only the female cohort. This indicates that there is likely a link between sex and risk of onset of KOA. Although the differences between the male and female only models are not significant, the small differences that are present highlight a potential need for a dedicated analysis with a larger sample size and more variables to consider how sex plays a role in the likelihood to develop and present with KOA. Further work should highlight additional features which could be masked by sex, such as sex specific cancers and their treatments, pregnancy, and hormone altering surgery such as hysterectomy and prostatectomy.

**Fig 5:**
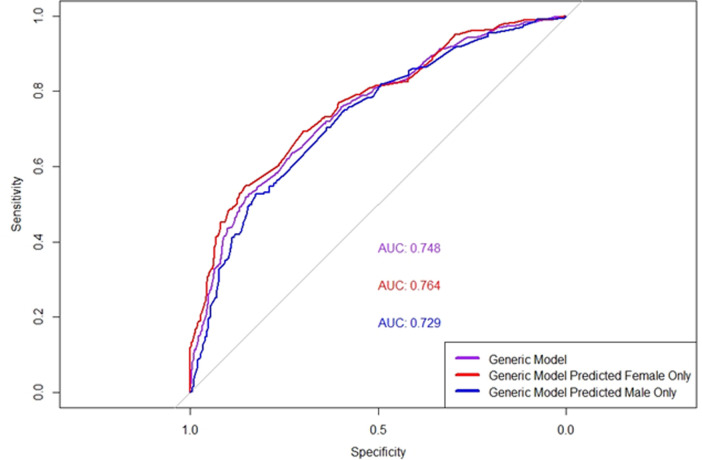
ROC curves for the generic model and the generic model tested on sex specific groups. ROC Curves Comparing the performance of the generic model on the whole cohort and sex-Specific groups. The AUC values are 0.764 for the predictions for females only, 0.748 for the overall Generic Model, and 0.729 for predictions on males only. Sensitivity and specificity are plotted to illustrate the trade-offs in classification performance.

The variables considered in the model from row 1 in Table 2 is the same as the model described in McCabe et al. [50]. The results in rows 2 and 3 are for the generic model from row 1 when tested individually on the male and female test cohorts, with the ROC curves for these shown in Fig 5.

**Fig 6:**
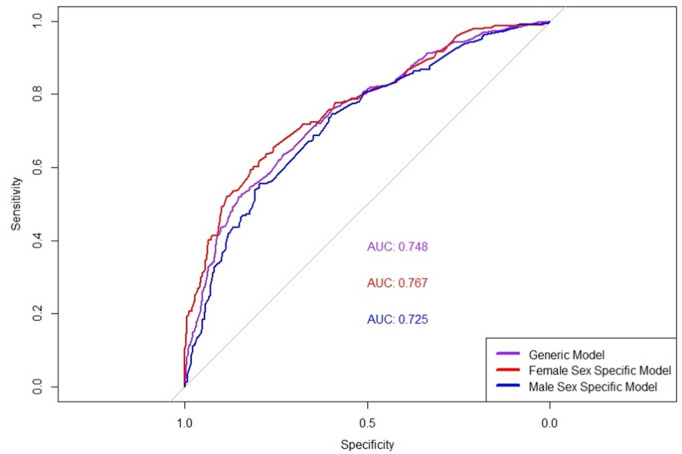
ROC curves for the generic model and the sex specific models for male and female. ROC Curves Comparing the Performance of Generic, Female-Specific, and Male-Specific Models. The AUC values are 0.767 for the Female-Specific Model, 0.748 for the Generic Model, and 0.725 for the Male-Specific Model. The graph highlights the sensitivity and specificity trade-offs in classification performance across different models.

Rows 4 and 5 detail the results for the curves shown in Fig 6. These models consider the original pool of variables plus the sex specific variables, and only those chosen by feature selection are included in the model.

**Fig 7:**
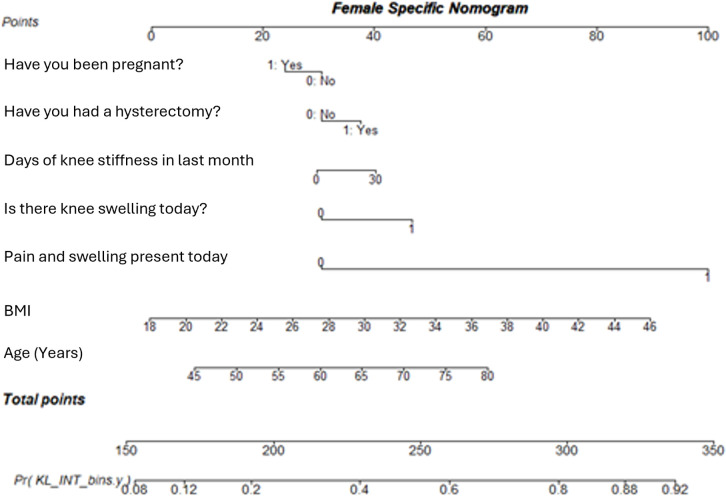
A nomogram showing the Female specific model for diagnosing KOA at first presentation. This model incorporates factors identified as significant predictors in females, providing a tailored risk assessment tool.

**Fig 8:**
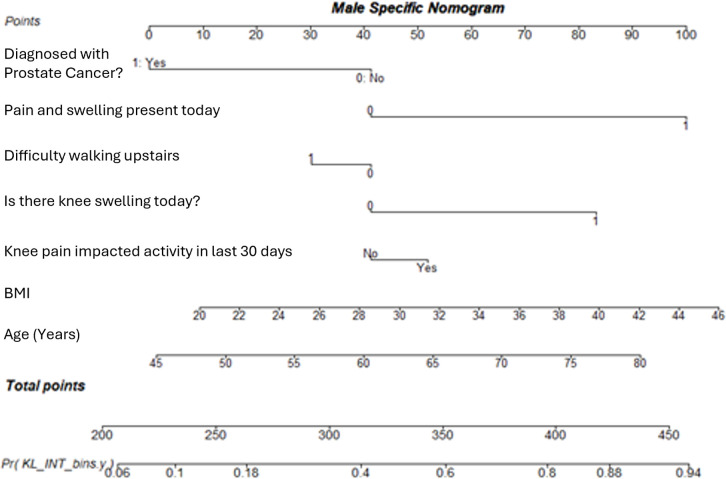
A nomogram showing the model calculated using the male specific variables to diagnose KOA at first presentation. The model is designed to account for male-specific risk factors and their contribution to KOA diagnosis.

It is clear from the sex specific nomograms (female in Fig 7 and male in Fig 8), that the variables contribute to the likelihood of having KOA differently when considered solely for males or females compared to the whole sample.

Considering Figs 7 and 8, BMI has a greater influence on a male’s likelihood to develop KOA than a female’s. Similarly, knee swelling in males has nearly double an increase in contribution to KOA, in comparison to females. Up to the age of 50, age contributes to female disease likelihood more than for males, but from age 55 years age contributes more to males, with 65 year old males having an equal age related contribution to the overall KOA likelihood as a 75 year old female.

**Fig 9:**
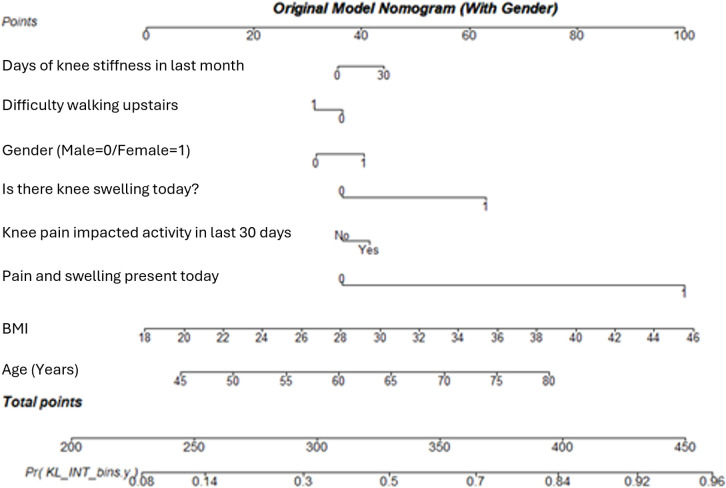
A nomogram displaying the variable contribution of the original diagnosis model, described in the paper by McCabe et al. [ 50], to the chance of having KOA at first presentation. The nomogram highlights the impact of each predictor variable on the overall likelihood of diagnosis.

Considering the generic model, displayed in Fig 9, there appears to be some difference between the variable contributions to likelihood for disease presence compared to the female specific and male specific models. This is supported by the AUC for these models ranging from 0.725 to 0.767, for the male and female specific models respectively, with the generic model in the range with an AUC of 0.748.

## Discussion

There are established disparities in the incidence of KOA associated with sex as well as an understanding that KOA manifests differently for both sexes, biologically and behaviourally [23]. Despite a growing pool of evidence that supports this finding, there is a lack of translation from this into practical implementations. This may lead to unconscious bias from healthcare professionals when diagnosing and identifying those at risk from KOA [28].

The sex-specific nomograms highlight distinct differences in how variables contribute to the likelihood of developing KOA between males and females. As previously discussed, BMI and knee swelling are variable indicators between males and females. These differences in variable contributions are supported by the AUC values, which show slightly higher predictive accuracy for the sex-specific models compared to the generic model. The results suggest that models incorporating sex-specific variables perform better than generic models, particularly for females. While the generic model serves as a benchmark, when applied to female data, there is a higher AUC than when applied to male data, and the McNemar test confirms statistical significance for females (p = 0.005). The female-specific model consistently outperforms the male-specific model, indicating that sex-disaggregated modelling can improve diagnostic accuracy, particularly for females.

Although the differences between male and female models are small, they underscore the need for further research with larger sample sizes to explore how sex-specific factors affect the likelihood of developing KOA. A deeper understanding into the mechanisms of how sex contributes to the likelihood of developing KOA in future work is necessary. Having a deeper understanding of sex specific mechanisms will allow for better management for individualised groups, leading to personalised and practical advice.

As far back as the 1980’s there have been more females affected by symptomatic KOA, but this has not influenced how disease in males and females is considered. A risk factor for KOA is being female, but when roughly half of the global population are female, this offers no comfort or insight into understanding the condition.

The studies carried out into the effect of sex on the overall benefits of surgical intervention show varying results, with no clear difference between males and females [62–64]. There is, however, evidence to show that after total knee arthroplasty, female patients are significantly less likely to be satisfied with the pain relief they experience [62].

Considering the female specific factors in the model, history of hysterectomy and history of pregnancy highlight a potential link to hormone involvement, specifically oestrogen. During pregnancy, the level of oestrogen in the body increases in comparison to the relative concentration in a woman who is not pregnant. After a hysterectomy the level of oestrogen in the body falls, a pattern that is mirrored in females who have gone through menopause. Studies focusing on whether oestrogen therapy has a protective effect for development of OA have conflicting findings, with some suggesting protective effects, and others suggesting the likelihood to increase risk to develop OA [65–67]. One study identified that hormonal and reproductive factors have an effect of risk of knee replacement [66], suggesting that consideration into these features should be accounted for when determining if someone is at risk of developing KOA.

Until further research is carried out into the effect of hormones, both male and female, into the likelihood of developing KOA there is likely to be an unintentional bias rooted in historical protocols that are still underpinning modern practices [68]. Studies have shown that there are genetic differences between males and females in every tissue in the body and until the effect of this is understood there needs to be a focus on engineering a ‘best-fit’ model for groups of the population, without overfitting to the trend highlighted in the data available at the time.

### Limitations

Despite the promising findings, there are several limitations to this study that must be considered. First, the dataset used may not fully represent the diversity of the population, potentially limiting the generalisability of the results to broader or more varied patient groups. The study was conducted in America, and caution must be used when extrapolating the findings to different groups that have different demographics within the population. The model’s reliance on sex-specific variables, while improving performance, could also risk oversimplifying the complexity of biological differences between sexes, as it may overlook other intersecting factors such as age, ethnicity, or socioeconomic status. Additionally, the model’s performance was primarily evaluated within a specific cohort, and further validation on independent datasets is needed to confirm its robustness across different settings and populations. Lastly, while the improved interpretability is a strength, there remains a need for ongoing efforts to ensure that the explanations provided by the model are clinically relevant and actionable, as some variables may still reflect surrogate factors rather than true causal mechanisms. These limitations suggest that further research is necessary to validate and refine the model before it can be widely applied in clinical practice.

## Conclusion

The analysis in this paper shows that there is scope for further investigation into the differences from a diagnostic point when considering the way KOA affects males and females, with the addition of sex specific information having potential clinical benefit to females. Although there were slight differences in the performance of the models when considering males and females separately, the overlap of the confidence intervals suggests that there is no real significance in the differences between the models. However, a McNemar test comparing the predictions for each sex specifically shows clear statistical significance for females (p = 0.005). This confirms that the female sex specific model performs better in the cohort after including additional sex specific variables. The additional information leveraged by using sex specific variables also indicates the importance of interpretability, as it may have uncovered underlying reasons for predictions instead of picking up surrogate factors that perform comparably.

This finding has broader implications for the development of predictive models in healthcare, as it highlights the importance of tailoring models to account for biological and demographic differences, such as sex. By including sex-specific variables, the model doesn’t just improve in performance, but it also enhances interpretability, which is critical for gaining clinical trust and insight into why certain predictions are made. This approach could potentially be extended beyond sex to include other demographic factors like age or ethnicity, further refining predictive models and ensuring they are more representative of diverse populations. In doing so, healthcare systems can address potential biases in AI-driven decision-making and promote more equitable and personalized care, ultimately leading to improved diagnostic accuracy, treatment efficacy, and patient outcomes across a wider spectrum of individuals.

Although models containing sex specific variables do not outperform the generic model, the factors in the sex specific models are more specialised, so therefore could arguably describe the difference in prediction performance. There is a statistically significant result in the prediction of female KOA according to the model presented in this paper. Although the sex specific models are not significantly different from the generic ones, they are more specific, which in a screening scenario could be more useful to both the clinician and the subject. One of the key ideas within this work is that of interpretability, and this additional information adds a layer of interpretability to the model that can help the subject better understand their condition. The added insight can be provided visually by using nomograms, and in this case, can also justify the advantage of using sex specific models as part of screening and patient education programmes.
